# A Typology of Work-Related Psychological Wellbeing Among Clergy: The Francis Burnout Inventory

**DOI:** 10.1007/s10943-026-02758-4

**Published:** 2026-09-25

**Authors:** Andrew Village, Leslie J. Francis

**Affiliations:** 1 School of Humanities, York St John University, York, UK; 2 Centre for Research in Intellectual and Developmental Disabilities (CIDD), University of Warwick, Coventry, UK; 3 World Religions and Education Research Unit, Lincoln Bishop University, Lincoln, UK

**Keywords:** Clergy, Burnout, Typology, Balanced affect, Validity, Wellbeing

## Abstract

The balanced affect model of clergy work-related psychological wellbeing operationalised in the Francis Burnout Inventory (FBI) maintains that professional burnout is predicted by the interaction between negative affect (emotional exhaustion) and positive affect (professional satisfaction). In order to locate negative affect and positive affect alongside one another, a five-fold typology was created using scores from 505 ministers in the Church of Scotland on the two scales of the FBI, the Satisfaction in Ministry Scale (SIMS) and the Scale of Emotional Exhaustion in Ministry (SEEM). The five types were characterised as Creaking (high SEEM and low SIMS scores), Coasting (low SEEM and low SIMS scores), Central (average SEEM and average SIMS scores), Compensating (high SEEM and high SIMS scores), and Cruising (low SEEM and high SIMS scores). The construct validity of this five-fold typology was demonstrated by comparing scores on three measures of the hypothesised consequences of burnout: the Warwick Edinburgh Mental Well-Being Scale, the Purpose in Life Scale, and the frequency of thinking about leaving ministry. On these measures, Creaking types showed the greatest evidence of burnout and Cruising types the least, with other types falling between these two groups. Subsequently, mean and standard deviations scores from the combined data from 8 surveys totalling 4658 clergy were used to indicate cut-off points for this five-fold typology to be employed in future studies.

## Introduction

### Introducing the Francis Burnout Inventory

The Francis Burnout Inventory (FBI) rests on a very different conceptual framework from that employed by the Maslach Burnout Inventory (MBI; Maslach & Jackson, 1986). The MBI construed burnout as a three-stage progressive experience. Stage one is experienced as emotional exhaustion (I feel emotionally drained by my work). High scores of emotional exhaustion give rise in stage two to depersonalisation (this job is making me uncaring). High scores of depersonalisation gives rise in stage three to low scores of personal accomplishment (I cease to agree that I accomplish worthwhile things in this job). What this model fails to explain is why some individuals simultaneously record high scores of emotional exhaustion *and* high scores of personal accomplishment.

By way of contrast, the FBI was rooted in Bradburn’s (1969) classic work on balanced affect and construed burnout as high emotional exhaustion (negative affect) alongside low personal accomplishment (positive affect). According to this account, negative affect and positive affect are not opposite ends of a single continuum, but operate (at least partially) as two separate continua. According to this account, positive affect holds the capacity to mitigate the deleterious consequences of negative affect. While the MBI is a generic measure designed to assess burnout across the people-facing profession, the FBI was developed specifically to assess positive affect and negative affect among clergy and religious professionals. In this context, negative affect was operationalised by the Scale of Emotional Exhaustion in Ministry (SEEM) and positive affect was operationalised by the Satisfaction in Ministry Scale (SIMS).

After several small-scale initial trials, Francis, Kaldor et al. (2005) field tested the Francis Burnout Inventory as part of the 2001 National Church Life Survey carried out by NCLS Research and partner organisations in Australia, England and New Zealand. In parallel with an attender survey, the *senior* minister/pastor/priest in each organisation was invited to complete a leader survey. Useful completed leader surveys were received from 6680 leaders across the three nations. The sample comprised 5496 men and 1143 women; 3903 from Australia, 2163 from England, and 614 from New Zealand. In this foundation study, Francis, Kaldor, et al. (2005) reported good internal consistency reliability for SEEM (*α* = .84) and for SIMS (*α *= 0.84).

The English language instrument developed by Francis, Kaldor, et al. (2005) has been employed in a range of studies, including work reported by Francis, Wulff, and Robbins (2008), Francis Robbins, et al. (2009), Robbins and Francis (2010), Brewster et al. (2011), Francis, Gubb, and Robbins (2012), Robbins et al. (2012), Barnard and Curry (2012), Francis, Robbins, and Wulff (2013a, 2013b), Francis, Payne, and Robbins (2013), Randall (2013a, 2013b, 2015), Tanner et al. (2013), Royle et al. (2014), Robbins and Francis (2014), Francis, Laycock, and Brewster (2015), Sterland (2015), Durkee-Lloyd (2016), Francis, Emslie, and Payne (2019), Francis, Village, and Payne (2021), Cordeiro et al. (2021), Swaminathan (2022), Francis, Haley, and McKenna (2023), Francis, Sailer, et al. (2024), Francis, Smith, and McKenna (2024), Smith et al. (2025), Frederick et al. (2024), Utomo et al. (2024), Rowe et al. (2024), and Kansiewicz et al. (2026). Research employing the FBI has now been extended to other linguistic and cultural contexts, including Italy (Francis & Crea, 2015), Portugal (Fabri et al., 2025), Brazil (Fabri et al., 2026), and Spain (Picornell-Gallar & González-Fraile, 2026).

The balanced affect approach to conceptualising and assessing vulnerability to burnout among clergy carries practical and pastoral implications for ways in which burnout is then managed. From the theoretical framework it is possible to distil recommendations for therapeutic and preventative interventions. The case is argued that it is more realistic to implement strategies that may enhance positive affect (satisfaction in ministry) than to remove the embedded triggers of negative affect (emotional exhaustion in ministry). Because of the importance of these practical implications for therapeutic and preventative interventions a series of studies has been designed to test this model. In particular three strands of research have examined different aspects of the claim made by the balanced affect model.

The first strand has examined the factor structure of the 22 items comprising the two scales of the FBI. While initial studies supported the factor structure of all 22 items, subsequent studies have demonstrated that a stronger solution can be generated by omitting one item from each scale (see Francis, Crea, & Laycock, 2021). On the basis of this analysis the revised (20 item) FBI is now to be preferred.

The second strand of research has supported the construct validity of the independence of the two measures of positive affect and negative affect by demonstrating that individual differences in the two measures may be explained by different predictor variables. For example, one trend emerging from these studies concerns the different associations with age. While scores on the measure of positive affect (satisfaction in ministry) are not related with age, scores on the measure of negative affect (emotional exhaustion) decrease with age. This is evidenced by recent studies among Catholic priests in Italy (Crea & Francis, 2022), Methodist ministers in Britain (Francis, Village, & Haley, 2023), and Catholic priests in Portugal (Fabri et al., 2025). A second example of different associations is provided by Crea and Bampoyiki (2025) who found that religious coping strategies were associated with enhanced positive affect (satisfaction in ministry) but were unrelated with negative affect (emotional exhaustion in ministry). A third example is provided by examination against the three subclinical pathologies of Machiavellianism, narcissism, and psychopathy as operationalised by the Short Dark Triad (SDT; Jones & Paulhus, 2014). The intriguing finding is that Narcissism is positively correlated with both negative affect and positive affect. In other words, higher narcissism scores are associated with higher scores of emotional exhaustion in ministry and higher scores of satisfaction in ministry, a clear indication that positive affect and negative affect are not functioning as opposite poles of a single continuum (Francis & Crea, 2021).

The third, and possibly most important strand of research has supported the core theory that increased levels of positive affect mitigate the deleterious consequences of negative affect. This third strand of research requires the identification of an outcome (dependent) variable (or set of variables) that may approximate to an hypothesised deleterious consequence of poor work-related psychological wellbeing or burnout. Multiple regression is then employed to examine the cumulative effects of negative affect (SEEM), positive affect (SIMS), and the interaction of negative affect and positive affect on that dependent variable. So far nine studies have employed this model of analysis among different groups of clergy and employing a range of different dependent variables. These studies have drawn on samples of: 744 clergy serving in the Presbyterian Church (USA) (Francis, Village, et al., 2011), 658 Anglican clergy serving in the Church of England (Francis, Laycock, & Brewster, 2017); 155 Catholic priests serving in Italy (Francis, Laycock, & Crea, 2017); 95 Catholic priests and 61 Catholic religious sisters serving in Italy (Francis, Crea, & Laycock, 2017); 358 Anglican clergy serving in the Church in Wales (Village, Payne, & Francis, 2018); 90 Anglican clergy serving in the Church of England (Francis, Laycock, & Ratter, 2019); 287 Catholic priests serving in Italy (Francis, Crea, & Laycock, 2021); 803 Methodist ministers serving in Great Britain (Francis, Village, & Haley, 2023); and 266 Catholic priests serving in Portugal (Fabri et al., 2025). Collectively these nine studies have each supported the balanced affect approach to assessing clergy work-related psychological wellbeing or burnout.

In terms of dependent variables, the most frequently used measure has been an index concerning thoughts of leaving ministry (Fabri et al., 2025; Francis, Laycock, & Brewster, 2017, Francis, Village, & Haley, 2023; Village et al., 2018), often alongside other pertinent rating scales. Two particular scales have also been employed in this series of studies. Francis, Laycock, and Crea (2017), Francis, Crea, and Laycock (2017), and Francis, Crea, and Laycock (2021) employed the Purpose in Life Scale developed by Robbins and Francis (2000). Francis, Laycock, & Ratter (2019) employed the Warwick-Edinburgh Mental Wellbeing Scale (Tennant et al., 2007).

### Person-Centred Approaches to Wellbeing

Quantitative measures on continuous scales are useful for researchers because they can indicate small differences in traits or symptoms between individuals. They also carry more statistical information than simpler, categorical measures. However, they are less useful for practical or personal use because it can be hard to get an overall profile without knowing how to interpret scale scores and being able to understand how scores on different measures interact. This had led some researchers to try and convert trait scores on personality models such as the Big Five into person-centred profiles (Asendorpf, 2015; Asendorpf & Denissen, 2006; De Fruyt et al., 2002). Creating a typology from continuous FBI scale scores makes theoretical sense because the balanced affect model is premised on the idea that positive affect can offset the deleterious effects of negative affect. So having high positive and high negative affect, or low positive and low negative affect, may point to a different ministry experience from having low scores in one affect and high scores in the other. Creating a typology of this nature also makes sense because the FBI was created primarily to help ministers improve their overall work-related psychological wellbeing. The first step in helping ministers to do that is to illuminate what different levels of positive and negative affect might mean in terms of overall ministry experience.

There have already been some attempts to create typologies from the Maslach Burnout Inventory (Mind Garden, 2018). In the original MBI manuals it was suggested that the three scales of emotional exhaustion, depersonalisation, and reduced personal accomplishment could be divided into thirds to give ‘high’, ‘medium’ and ‘low’ scores for each. These had not been validated against actual levels of burnout, however, and the suggestion was removed from the fourth edition of the MBI manual (Maslach et al., 2016). Instead, a typology was suggested based on the work of Leiter and Maslach (2016), who used latent profile analysis to identify five profiles, which they termed ‘Burntout’, ‘Engagement’, ‘Overextended’, ‘Disengaged’, and ‘Ineffective’.

A recent qualitative study from the Clergy Health Initiative Longitudinal Survey (CHILS) of 1460 pastors from the United Methodist Church in North Carolina used latent class analysis to identify groups with similar profiles of mental health and wellbeing (Tice et al., 2025). The analysis was based on general measures of mental health and wellbeing, including the MBI. Follow-up interviews of pastors selected from the four identified groups suggested a four-fold typology with groups labelled ‘Flourishing’, ‘Burdened but Fulfilled’, ‘Languishing’, and ‘Distressed’. Although this work was not using a balanced affect model, the idea of a group of ministers being ‘Burdened but Fulfilled’ echoes the title of the paper that established the FBI: ‘Happy but exhausted? Work-related psychological health among clergy’ (Francis, Kaldor et al., 2005). This study builds on the idea of creating a typology from continuous variables by using the two scales of the FBI to group clergy according to their relative levels of positive affect and negative affect.

### Research Aims

Against this background the present study has four specific research aims drawing on a previously unpublished set of data provided by 505 ministers serving in the Church of Scotland who completed the Francis Burnout Inventory alongside measures of purpose in life and mental wellbeing. These aims are:

- To test the internal consistency reliability of the two measures of satisfaction in ministry (positive affect) and emotional exhaustion in ministry (negative affect) among this population
- To test the theory of balanced affect among this population employing the measure of purpose in life as the dependent variable
- To propose and test a typology of work-related psychological wellbeing among this specific population
- To employ this newly developed typology to locate this specific group of clergy (ministers serving in the Church of Scotland) against the mean scores recorded on the two scales of the Francis Burnout Inventory by diverse groups of clergy.

## Method

### Procedure

In 2017 a survey among ministers serving in the Church of Scotland was commissioned by the General Assembly as part of its commitment to support those engaged in ministry. The survey was sent by post under a covering letter from the Moderator of the Church of Scotland and returned by post to the research group in Wales. Participation was voluntary, anonymous, and confidential. The survey included sections on: time spent on ministry functions; feelings about ministry, life and faith; physical and spiritual wellbeing and mental health; and role responsibilities and career paths. A total of exactly 1000 copies of the survey were mailed. Of these ten were returned because the recipient had moved, had retired, or was on long-term sick leave, and 505 were returned fully completed, generating a response rate of 51%. Some others were returned blank or only partly completed.

### Participants

Of the 505 ministers who returned fully completed surveys, 337 were male, 165 female and three individuals who preferred not to disclose their sex; the majority were aged fifty or over, with two in their twenties, 17 in their thirties, 81 in their forties, 204 in their fifties, 168 in their sixties, 31 in their seventies, and two individuals who preferred not to disclose their age.

### Instruments

The Francis Burnout Inventory (FBI) originally comprised two 11-item scales, the Scale of Satisfaction in Ministry (SIMS) and the Scale of Emotional Exhaustion in Ministry (SEEM) (Francis, Kaldor, et al., 2005). The scale was later revised to two 10-item scales by removing one item from each scale, which improved the factor structure (Francis, Crea, & Laycock, 2021). Each item is rated on a five-point Likert scale ranging from disagree strongly (1) to agree strongly (5). Example items concerned with satisfaction in ministry are: ‘The ministry here gives real purpose and meaning to my life’ and ‘I gain a lot of personal satisfaction from working with people in my current ministry’. Example items concerned with emotional exhaustion are: ‘I feel drained in fulfilling my ministry roles’ and ‘I am less patient with those among whom I minister than I used to be’. Alpha reliabilities for the 10-item scales in a sample of 287 Catholic priests and religious sisters in Italy were .78 for SEEM and .84 for SIMS (Francis, Crea, & Laycock, 2021).

A range of psychological instruments in the survey were used as dependent variables to validate the four FBI types:

*The Warwick Edinburgh Mental Wellbeing Scale (WEMWBS)* consists of 14 items that measure both hedonic and eudaimonic aspects of mental health such as positive affect, interpersonal relationships, and positive functioning (Tennant et al., 2007). It has been shown to have good reliability and construct validity in a range of populations (Stewart-Brown, 2013). Cronbach alpha reliability for this sample was .92.

*The Purpose in Life Scale (PILS)* consists of 12 Likert items with a five-point response scale (Robbins & Francis, 2000). It includes items such as ‘My personal existence is full of meaning’ and ‘I feel my life is going nowhere’ and is coded such that a high score indicates a positive purpose in life. The scale has been tested in a range of samples and shown to have good to excellent internal consistency reliability (see Francis, Crea, & McKenna, 2019 and references therein). Cronbach alpha reliability for this sample was .92.

*Leaving ministry* items have been used to assess the validity of the FBI among clergy samples (Francis, Village, & Haley, 2023; Francis, Village, et al., 2011) on the assumption that this is a proxy measure for the consequences of burnout. The survey included the question ‘Have you ever considered leaving ministry?’ with possible responses of ‘No’, ‘Once or twice’, and ‘More often’.

### Analysis

The first step was to examine the properties and relationship of the two scales of the FBI. The second step was to create the typology and calculate the frequency of each FBI type in the sample. The typology was theory driven rather than data driven: mean scores on the two scales of the FBI were used to create a four-fold typology, which was amended by creating a fifth group that had scores with half a standard deviation of the mean of both scales. The third step in analysis was to compare mean WEMWBS and PILS scores between the five types. Results in this section are presented graphically (mean ± 95% CL). The final step in analysis was to compare the leaving ministry measure across FBI types using contingency table analysis.

## Results and Discussion

### Examining the Measures

Table 1 explores the scale properties of the two measures proposed by the Francis Burnout Inventory (FBI), the Scale of Emotional Exhaustion in Ministry (SEEM) and the Satisfaction in Ministry Scale (SIMS) in terms of the correlations between the individual items and the sum of the other nine items in the scale and the item endorsements as the sum of the agree and agree strongly responses. In terms of SEEM, the item rest of test correlations ranged between .40 and .66. The item with the highest correlation (Fatigue and irritation are part of my daily experience) captures the heart of this construct. Percentage endorsement ranges from 13% who agreed that they were becoming less flexible in their dealings with those among whom they minister to 47% who agreed that they find themselves frustrated in their attempts to accomplish tasks that are important to them. In terms of SIMS, the item rest of test correlations ranged from .39 to .72. The item with the highest correlation (I gain a lot of personal satisfaction from fulfilling my ministry roles) captures the heart of the construct. Percentage endorsements ranges from 53% who agree that they deal very effectively with the problems of the people in their current ministry to 88% who gain a lot of personal satisfaction from working with people in their current ministry.

**Table 1:** Francis Burnout Inventory: scale properties

	*r*	%
*Scale of Emotional Exhaustion in Ministry*		
I feel drained by fulfilling my ministry roles	.62	43
Fatigue and irritation are part of my daily experience	.66	41
I am invaded by sadness I can’t explain	.55	18
I am feeling negative or cynical about the people with whom I work	.61	16
My humour has a cynical and biting tone	.40	16
I find myself spending less and less time with those among whom I minister	.42	31
I have been discouraged by the lack of personal support for me here	.52	19
I find myself frustrated in my attempts to accomplish tasks important to me	.58	47
I am less patient with those among whom I minister than I used to be	.52	22
I am becoming less flexible in my dealings with those among whom I minister	.48	13
*Satisfaction in Ministry Scale*		
I have accomplished many worthwhile things in my current ministry	.52	75
I gain a lot of personal satisfaction from working with people in my current ministry	.54	88
I deal very effectively with the problems of the people in my current ministry	.44	53
I feel very positive about my current ministry	.71	69
I feel that my pastoral ministry has a positive influence on people’s lives	.40	86
I feel that my teaching ministry has a positive influence on people’s faith	.39	68
I feel that my ministry is really appreciated by people	.59	78
I am really glad that I entered the ministry	.56	85
The ministry here gives real purpose and meaning to my life	.66	78
I gain a lot of personal satisfaction from fulfilling my ministry roles	.72	83

*r* = correlation between individual items and the sum of the remaining items

% = sum of agree and agree strongly responses

**Fig. 1:**
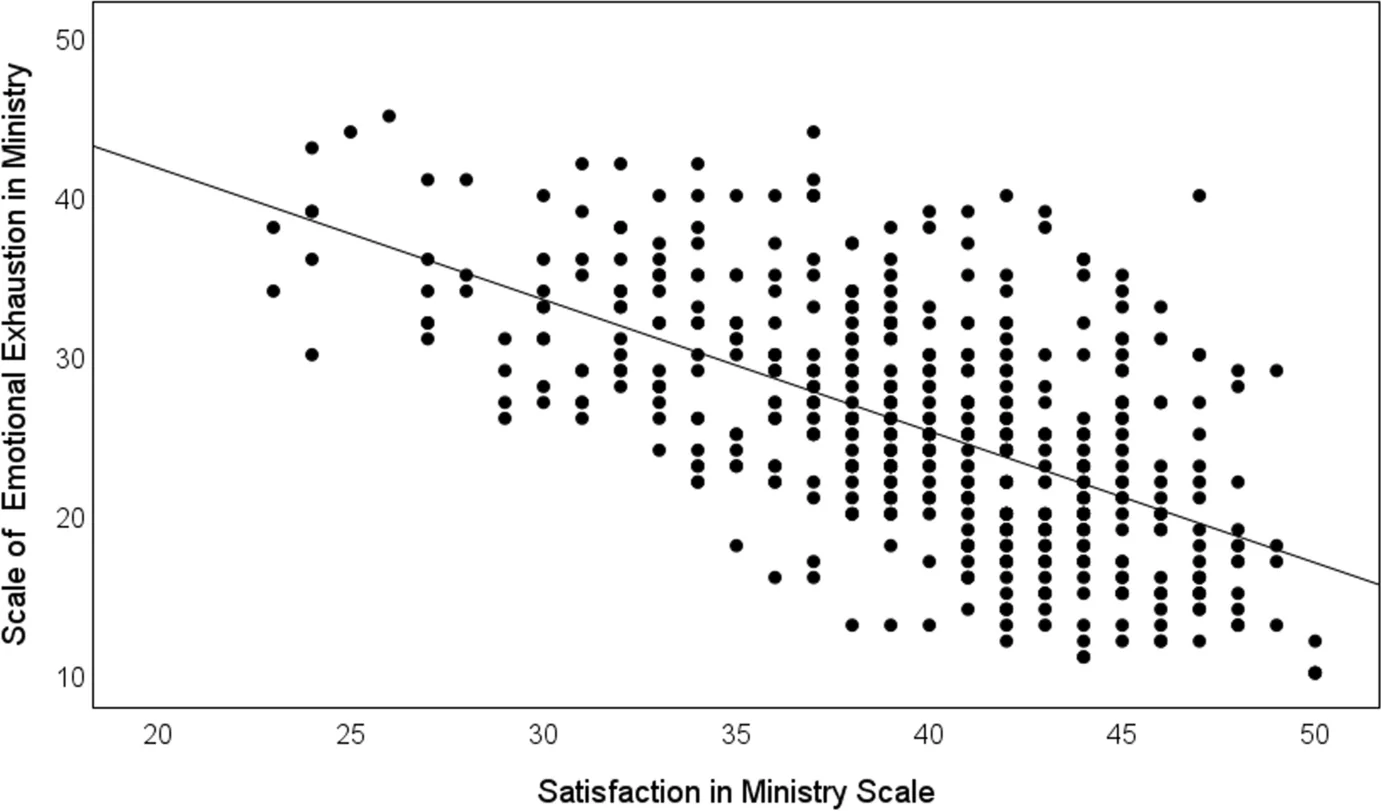
Regression of SEEMS on SIMS. *Note*. The line is the fitted linear regression y = − 0.83x + 58.3

Table 2 examines the scale properties of the four measures employed in the current study. All four report satisfactory levels of internal consistency reliability (Cronbach, 1951). Both scales of the FBI had the same potential range of scores (10–50), but mean scores for SEEM (25.4, SD = 7.3) were lower than for SIMS (39.7, SD = 5.3). The two scales were negatively correlated (*r* = − .60, *df* = 503,* p* < .001), with SEEM scores declining on average by .83 for every one-point increase in SIMS score (Fig. 1). There was considerable scatter around the regression slope, with the coefficient of determination (R^2^) showing that 36% of the variance in SEEM scores was explained by SIMS scores.

**Table 2:** Scale properties

	Alpha	Mean	SD
Emotional exhaustion	.84	25.4	7.3
Satisfaction in ministry	.85	39.7	5.3
Purpose in life	.92	49.6	6.4
Mental wellbeing	.92	49.7	8.1

Table 3 tests the balanced affect model of work-related psychological wellbeing and burnout by examining the independent and interaction effects of scores recorded on SEEM and SIMS on scores recorded on the Purpose in Life Scale, after first controlling for the effects of sex and age on the dependent variable. The data indicated a significant interaction between SEEM and SIMS scores. The significant interaction terms suggest that the mitigating effect of satisfaction in ministry scores on purpose in life scores was greater when levels of emotional exhaustion were higher, thus supporting the construct validity of the balanced affect approach.

**Table 3:** Multiple regression of SEEM and SIMS on PILS

	β	SE	*t*	*p* <
Constant	40.238	6.530	6.18	.001
Sex	− 0.359	.444	− 0.81	NS
Age	− 0.303	.229	− 1.32	NS
SEEM	− 0.593	.200	− 2.96	.01
SIMS	.317	.150	2.47	.05
SEEM and SIMS	.011	.005	2.37	.05

### Creating the FBI Typology

The data from the 505 ministers in the Church of Scotland survey were initially used to create a four-fold typology. The balanced affect model, which underpins the FBI, lends itself to the creation of a typology based on theoretical grounds, rather than one derived by statistical analysis of data. The latter usually involve methods such as latent class analysis (for categorical observed variables) or latent profile analysis (for continuous observed variables, as is the case here). The algorithms are designed to identify clusters, and are ‘blind’ to any underpinning theory, so they may, in any particular set of data, generate groupings that are hard to interpret and of little practical use. Balanced affect inherently points to a basic four-fold typology that recognises ministers who may have above or below average scores on either of the scales, so this was the initial approach adopted here.

Mean SEEM and SIMS scores were used as group boundaries to categorise ministers into two groups on each scale resulting in four groups: high SEEM with low SIMS, low SEEM with low SIMS, high SEEM with high SIMS, and low SEEM with high SIMS. Creating groups in this way has advantages and disadvantages. On the one hand, it is easy to do, easy to understand, and having four groups makes it easier to analyse than having many groups. On the other hand, the nature of the distribution meant that a number of people who had fairly similar scores on both scales were assigned to different groups because their scores happened to fall one side or the other of the mean. This adds uncertainty as to which quadrant (type) they should fall into. For this reason, those with scores near the mean values of both SEEM and SIMS were reassigned to a fifth type, using plus or minus half a standard deviation as the criterion on each scale. Although the use of means and ± 0.5 SD is a somewhat arbitrary choice, it does offer a simple and robust technique for assigning types which works well with the kinds of regression distributions typically found for this instrument (see Fig. 1). Having a simple and easily applied set of criteria is important for an instrument that is primarily designed as a pastoral rather than research tool.

**Fig. 2:**
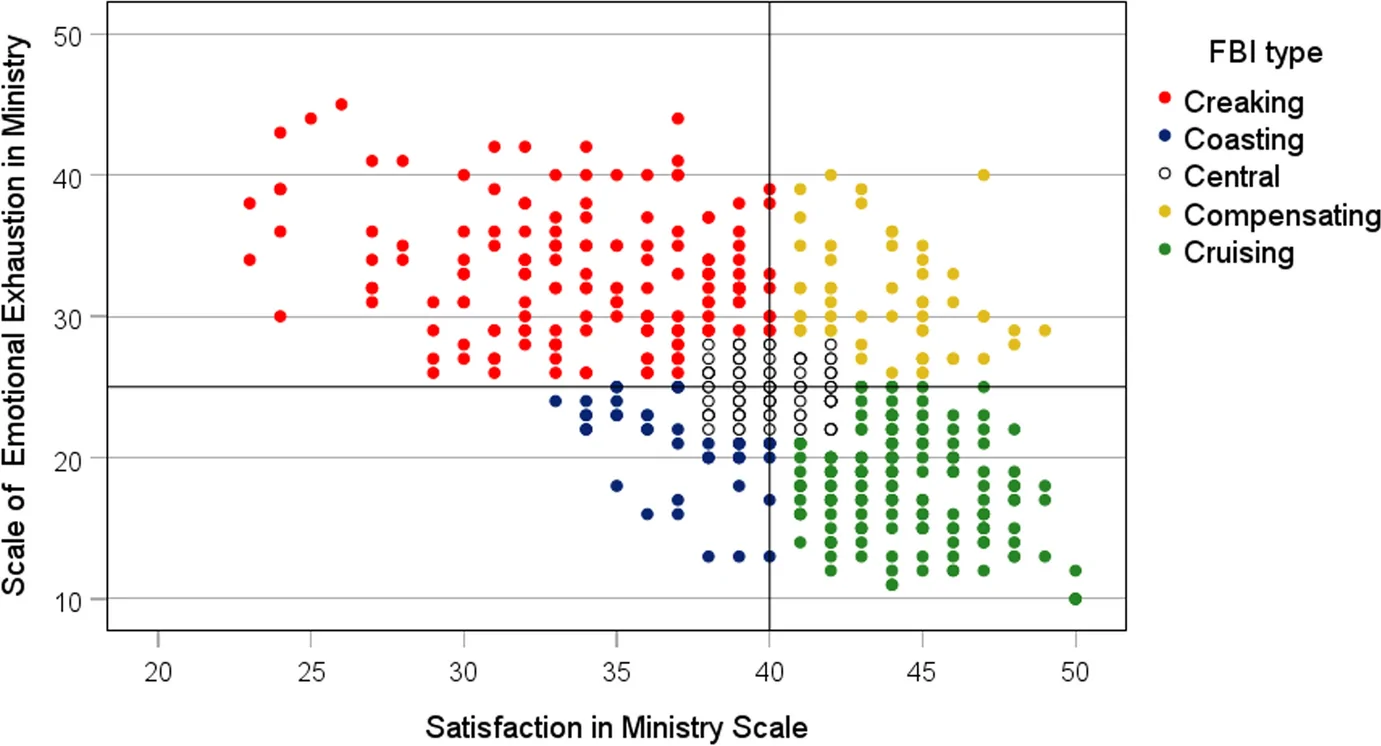
The five types of the FBI

The final typology is illustrated in Fig. 2, with each outer quadrant representing a different group and a central group in the middle of the distributions of both SEEM and SIMS. The five types were then named using terms that indicated what the balanced affect model might suggest about them in terms of exhaustion and satisfaction in ministry: Creaking, Coasting, Central, Compensating, and Cruising.

1. *Creaking*: those who were above average in exhaustion and below average in satisfaction. These are people who are likely to feel exhausted by what they do and who do not get much satisfaction from it either, so they are the most vulnerable to burnout. They may be creaking under the strain of what they are expected to do.
2. *Coasting*: those who were below average in both exhaustion and satisfaction. These are people who might feel a sense of professional disengagement related to them not having particularly heavy demands in their ministry but who, by the same token, may not get much satisfaction from their role either. They may be neither creaking nor cruising but instead fall somewhere between the two.
3. *Central*: those who have near-average scores for both exhaustion and satisfaction. Small changes in emotional exhaustion or satisfaction might move them into one of the other groups, but they may perceive themselves to be neither particularly exhausted nor particularly satisfied.
4. *Compensating*: those who were above average in both exhaustion and satisfaction. These are people who are likely to have a demanding and exhausting ministry, but who may relish the challenge and have a satisfying sense that they are achieving something. We may expect them also to fall somewhere between the Creaking and the Cruising types, but they may be a little different from the Coasting types.
5. *Cruising*: those who were below average in exhaustion and above average in satisfaction. These are people who are likely to feel little emotional exhaustion and who are generally satisfied with their ministry, so they are the least vulnerable to burnout. They find ministry relatively undemanding and something that they enjoy, so they look forward to meeting the challenges that each day brings.

Of the 505 ministers in the sample, just under a third were classed as Creaking, a similar proportion were classed as Cruising, just under one in ten were classed as Coasting, just over one in ten were classed as Compensating, and the remainder (just under one in five) were classed as Central (Table 4).

**Table 4:** Frequency of FBI types among Church of Scotland ministers

	SEEM	SIMS	Frequency	%
Creaking	Hi	Lo	157	31
Coasting	Lo	Lo	43	9
Central	Mid	Mid	89	18
Compensating	Hi	Hi	53	11
Cruising	Lo	Hi	163	32

N = 505; Hi = High, Lo = Lo, Mid = Midway

### Validating the FBI Typology

**Fig. 3:**
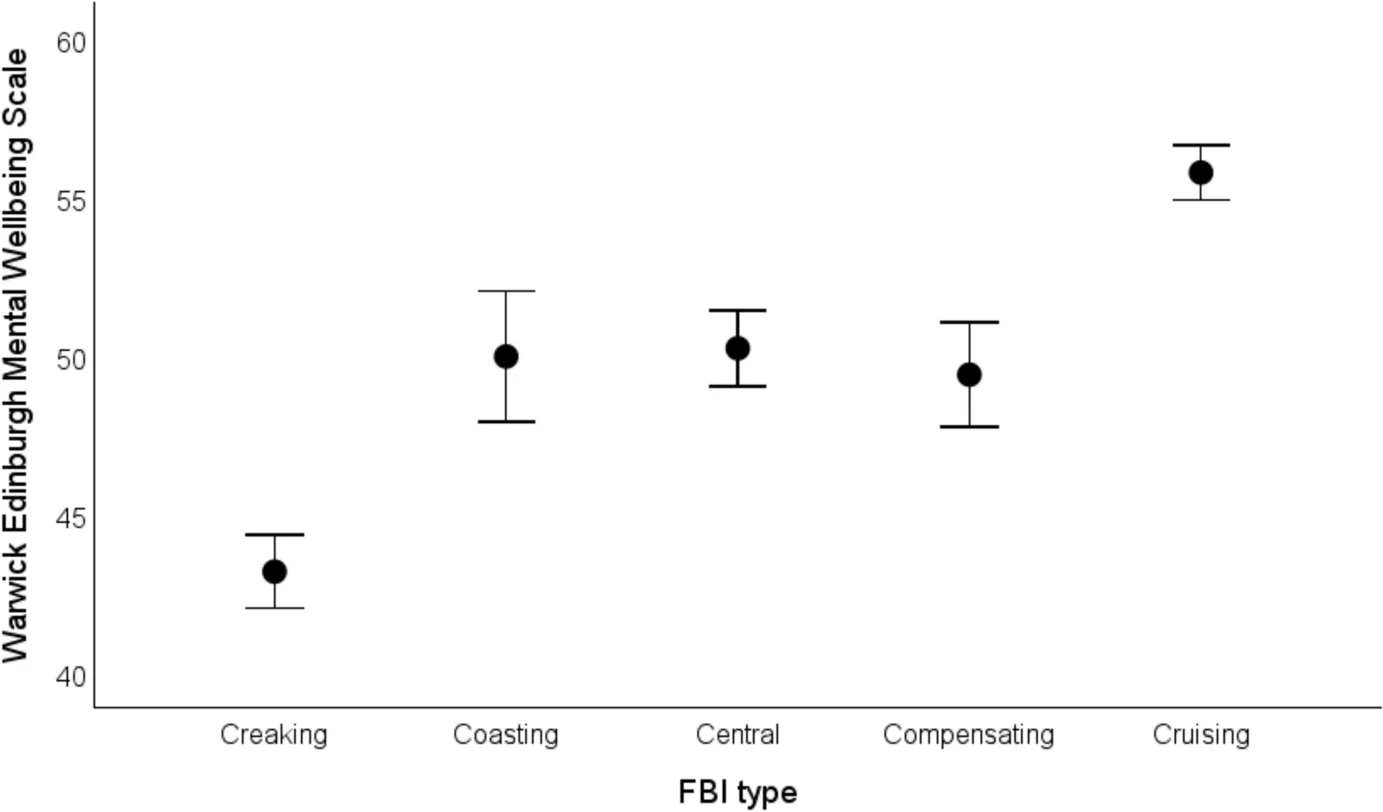
Warwick Edinburgh Well-Being Scale (WEMWBS) scores by FBI types. *Note *Error bars in Figs. 3 to 10 are 95% Confidence Limits

*The Warwick Edinburgh Wellbeing Scale (WEMWBS).* Creaking types scored lowest and Cruising types highest, with Coasting, Central, and Compensating types having similar scores which fell somewhere between the two (Fig. 3). The compensatory force of ministry satisfaction seemed to improve wellbeing significantly among the emotionally exhausted (Compensating types), but not to the levels of those who were satisfied and not stressed (Cruising types). Coasting types, who had lower than average satisfaction and lower than average emotional exhaustion, did better than Creaking types and had similar scores to Compensating types.

**Fig. 4:**
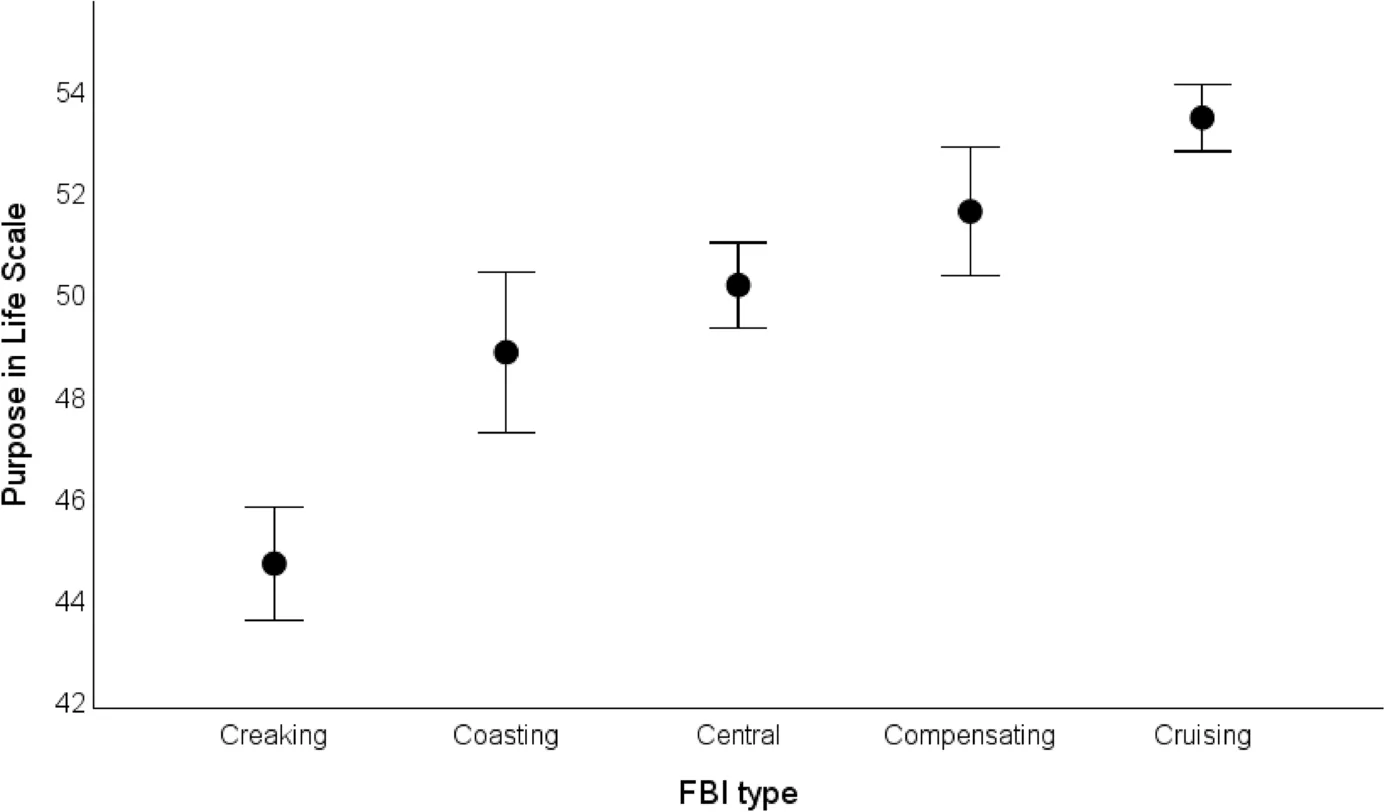
Mean (95% CL) Purpose in Life Scale Scores by FBI type

*Purpose in Life Scale (PILS).* The difference between types was similar to the WEMWBS, with Creaking types scoring lowest and Cruising types highest, with Coasting, Central, and Compensating types having similar scores which fell somewhere between the two (Fig. 4).

*Leaving ministry.* The frequency of considering leaving ministry varied significantly between the five FBI types (Table 5). Nearly three-quarters (71%) of Creaking types had considered leaving ministry at least once, compared to only 28% of Cruising types (Table 5). Coasting (40%), Central (52%) and Compensating types (55%) fell between these two. Compensating seems to reduce the chances of leaving, not as much as those who have low exhaustion as well as high satisfaction but slightly more than those who have low exhaustion and low satisfaction, who may leave because they are less engaged.

**Table 5:** Considered leaving ministry and FBI types

	Creaking	Coasting	Central	Compensating	Cruising	Total
N=	157	43	89	53	163	505
	%	%	%	%	%	%
No	29	60	48	45	73	51
Once or twice	42	28	45	40	22	34
More often	29	12	7	15	6	15

### Grounding the FBI Typology

So far, the rationale for creating the FBI typology has been tested and validated on a single group of clergy: 505 ministers serving in the Church of Scotland. Church of Scotland ministers have been individually assigned to one of the five types on the basis of the mean scores established within this sample. Using sample means creates types that are relative to others in the group but does not give a sense of whether this particular ministry is more exhausting or satisfying compared with others. A better approach might be to use means generated by combining results from a wide range of ministers. Fortunately, there have been a number of studies of different groups of ministers using the FBI 11- or 10-item scales (Table 6). Eight datasets with a total 4658 ministers were reanalysed to create an overall means using the ten-item revised FBI. These means and standard deviations (SEEM: 26.1 (SD = 7.1); SIMS: 39.0 (SD = 5.7)) are recommended as the best current cut-off points for assigning ministers to FBI types based on their SEEM and SIMS scores (using plus or minus half a standard deviation on each scale to delimit the Central type).

**Table 6:** Previous studies using the FBI on English-speaking populations of clergy

			SEEM10		SIMS10	
Study	Population	N	Mean	SD	Mean	SD
1	Church of Scotland	472	25.8	7.4	39.6	5.5
2	Salvation army	445	29.1	7.3	36.7	6.9
3	Church of England curates	253	23.6	6.2	41.5	4.9
4	Anglican clergy	688	26.3	7.0	37.3	5.6
5	Methodist ministers GB	803	26.7	6.3	38.0	5.0
6	Church in wales	368	25.3	6.7	38.7	4.9
7	Presbyterian church USA	675	24.3	7.1	40.3	5.3
8	Church of England	954	26.5	7.3	40.6	5.4
	Total	4658	26.1	7.1	39.0	5.7

*Sources* 1: present study; 2 L.J. Francis unpublished data; 3 (Smith et al., 2025); 4 (Francis, Laycock, & Brewster, , 2017); 5 (Francis, Village, & Haley, , 2023); 6 (Village et al., 2018); 7 (Francis & Village, 2011); 8 A. Village unpublished data. Studies using the FBI 11-item scales were recalculated using the 10-item scales. Respondents aged over 69 were excluded from this analysis

The different profiles generated for each sample using either overall or within-sample means are shown in Table 7. Within-sample typologies tend to be similar to one another because, in general, the slopes and scatter of the SEEM-SIMS regressions show little variation between clergy in different contexts. What does vary are the mean scores for exhaustion or satisfaction. As a consequence, samples where means are close to the overall mean (for example, sample 8) tend to have a similar typology profile to that calculated from the overall mean. However, those with divergent means show more variation using overall means to calculate the typology, and this might be more helpful to those managing groups of clergy in a particular context. For example, sample 2 (Salvation Army) has the lowest satisfaction score and highest exhaustion score of the eight samples. Using within-sample means, 29% were Creaking and 34% were Cruising. Using overall means, the figures were 45% and 21% respectively, which highlights the much greater vulnerability to burnout in this group of ministers compared with others. Where means differ in the opposite direction, such as sample 3 (Anglican curates), the typology highlights the lower vulnerability of a group to burnout. In this case within-sample means led to 28% Creaking and 31% Cruising, while overall means led to 12% Creaking and 51% Cruising.

**Table 7:** Comparison between calculating typology using overall versus within-sample means for SEEM and SIMS

		Overall means					Within-sample means				
		Creak %	Coast %	Cent %	Comp %	Cruis %	Creak %	Coast %	Cent %	Comp %	Cruis %
1	Church of Scotland	27	9	15	12	37	29	7	19	12	34
2	Salvation Army	45	9	13	12	21	29	8	16	13	34
3	Church of England curates	12	7	19	12	51	28	11	17	13	31
4	Church of England	35	16	20	4	25	28	7	23	10	33
5	Methodists Great Britain	33	11	25	7	24	29	8	20	11	33
6	Church in Wales	26	13	22	8	31	26	9	23	11	31
7	Presbyterian Church USA	22	8	14	8	47	30	10	20	9	33
8	Church of England	23	5	17	17	39	26	8	22	12	32

For details of studies, sample sizes, and means, see Table 6. Creak: Creaking; Coast: Coasting; Cent: Central; Comp: Compensating; Cruis: Cruising

### Limitations

Limitations with the present study are noted both within the specific study of Church of Scotland ministers on which the typology was established and within the group of studies that generated the overall means. The Church of Scotland study included a limited range of indices indicative of the deleterious consequences of burnout, namely a single item measure of thoughts of leaving ministry and two scales measuring purpose in life (Robbins & Francis, 2000) and mental wellbeing (Tennant et al., 2007). Future studies could address this limitation by including a richer range of indices. The overall means were calculated on just eight studies (including the Church of Scotland study), totalling just 4658 clergy. These overall mean scores can (and should) be recalculated as other comparable studies come to hand.

## Conclusion

The balanced affect model of clergy work-related psychological health predicts that the positive affect gained from satisfaction with ministry may partly offset the negative affect associated with emotional exhaustion in ministry. This implies that ministers with high levels of exhaustion and high levels of satisfaction may be less likely to exhibit evidence of burnout than those with equally high exhaustion but lower satisfaction. A sample of 505 Church of Scotland ministers was used to test this idea by creating a typology based on whether individuals were above or below the mean scores on the two scales of the Francis Burnout Inventory (FBI), the Scale of Emotional Exhaustion in Ministry (SEEM) and the Satisfaction in Ministry Scale (SIMS). A fifth group was identified as those whose scores were within half a standard deviation of the means of both scales. As predicted, ministers with high exhaustion and low satisfaction had the lowest average scores on the Warwick Edinburgh Mental Wellbeing Scale (WEMWBS) and the Purpose in Life Scale (PILS) and were most likely to have thought of leaving ministry at least once. Ministers who had low exhaustion and high satisfaction had the highest average scores on the WEMWBS and PILS and were least likely to have thought of leaving ministry. Those who had high exhaustion and high satisfaction, who had low exhaustion and low satisfaction, or whose scores were near the middle of both scales, had wellbeing and purpose in life scores that fell between the other two groups. This suggests that there may be some compensation happening in the high exhaustion and high satisfaction group.

The five groups were given names based on the theory underlying the balanced affect model: Creaking, Coasting, Central, Compensating, and Cruising. This was partly because the purpose of a typology is to allow a person-centred approach to burnout that could help practitioners as well as researchers. The validation of the typology using measures that are related to burnout suggests that types at either end of the typology (Creaking and Cruising) exhibit significantly different vulnerability of burnout from each other, and that other types fall somewhere between these two. Between them, Creaking and Cruising accounted for 62% of the ministers in this sample. Using SEEM and SIMS scores to identify these two groups is worthwhile in itself, but there was also evidence that the typology may be useful in pointing to ministers who fall in the minority categories of Coasting, Central, and Compensating. WEMWBS scores for these three groups were similar to one another and fell between the other two. In this case, hedonic and eudemonic wellbeing may ultimately be similar whether ministers have undemanding but unsatisfying jobs or demanding but satisfying jobs.

There was some suggestion that, as the theory predicts, Compensation types (with high exhaustion and high satisfaction) were closer to Cruising types than either the Coasting or Central types. This is crucial for therapeutic use of the FBI because it points to the value of satisfaction in ministry as a counter to the inevitable exhaustion that comes with it.

It is important to recognise the nature of this typology as a tool for assessing vulnerability to burnout among ministers in their current ministry context, rather than as a clinical diagnostic tool or as a method for categorising people along the lines of, say, psychological type models. Although personality and pathologies might predispose ministers to exhaustion or satisfaction in their roles, burnout is the interaction of a practitioner operating in a particular capacity, and the FBI typology is designed as a workable method of identifying whether and how vulnerability to burnout is operating at a given moment.

Calculating types using overall means calculated from a wide range of ministers, rather than those from a particular sample, has the advantage of allowing ministers to see how they compare to ministers generally, rather than their particular cohort. There may be times when the latter is useful for therapeutic work within a particular group of ministers, but we would recommend using overall means in most cases. Differences within samples will still be apparent, but groups that are generally more vulnerable to burnout will be more easily identified using overall means. More work is needed on a wider range of samples to define the overall means and cut-off points, and with more measures of the consequences of burnout to see if the evidence of affect balance shown in this study holds more widely. In particular, it would be good to know if ministers who are assigned to types on the basis of their SEEM and SIMS scores feel that the descriptions of their FBI type match the reality of their professional life.
